# Post-Traumatic Outcomes among Survivors of the Earthquake in Central Italy of August 24, 2016. A Study on PTSD Risk and Vulnerability Factors

**DOI:** 10.1007/s11126-021-09908-9

**Published:** 2021-05-11

**Authors:** Olimpia Pino, Annalisa Pelosi, Valentina Artoni, Massimo Mari

**Affiliations:** 1 Department of Medicine and Surgery, University of Parma, Via Volturno n. 39, 43125 Parma, PR Italy; 2 Department of Mental Health, Extensive Area 2, ASUR, Via dei Colli, 60035 Jesi, Italy

**Keywords:** PTSD symptomatology, Natural disasters, Stress, Anxiety, Depression

## Abstract

Central Italy suffered from the earthquake of 2016 resulting in great damage to the community. The purpose of the present study was to determine the long-term traumatic outcomes among the population. A preliminary study aimed at obtaining the Italian translation of the first 16 item of HTQ IV part [1] which was administered, 20 months after the disaster, at 281 survivors. In backward stepwise logistic regressions models, we estimated among the respondent’s characteristics and event-related variables the best predictors of Post-Traumatic Stress Disorder (PTSD).

A Confirmatory Factor Analysis (CFA) revealed a HTQ five-factors solution as best model, with satisfactory indexes of fit. HTQ held a positive correlation with both the SQD-P (r = .65, *p* < .05) and SQD-D subscales (r = .47, p < .05). ROC analysis suggested an area of .951 (95% CI = .917–.985) for the PTSD prediction. Basing on sensibility (.963) and specificity (.189), the best cut-off of 2.0 allowed discriminating for PTSD positive cases. After 20 months of the earthquake, the estimate prevalence of PTSD among the survivors is of 21.71% with a consistent and graded association between exposure variables and vulnerability factors (gender, age, exposure to death and home damage) and PTSD symptoms.

## Introduction

Earthquakes are unpredictable, uncontrolled disasters that cause widespread devastation threatening physical and psychological integrity of affected populations. Italy is one of the most seismically active countries in Europe. These disasters are associated with severe psychiatric disorders among survivors such as Major Depressive Episode (MDE), Generalized Anxiety Disorder (GAD), Panic Disorder, Obsessive-Compulsive Disorder (OCD), Post-Traumatic Stress Disorder (PTSD), specific phobias for loud sounds (thunders or thunderstorms), “quick trigger” suicidal episodes, substance abuse, and impaired quality of life [2–4]. The most distinctive condition is the Post-Traumatic Stress Disorder (PTSD), frequently co-occurring with depression, with prevalence rate of 23.66%, as recently calculated [5] estimating that 17.706 individuals resulted affected by PTSD from the total of 76.101 survivors exposed to seismic events from 1999 to 2013. This disorder is described in the DSM-5 [6] as a group of symptoms, which is triggered by life-threatening events lasting for more than a month from the direct or indirect exposure to a terrifying event implying real or threatened death, serious injuries, or sexual violence. Moreover, in order to fulfill criteria for the PTSD diagnosis, symptoms from four groups (clusters) must be arisen: (1) persistent re-experiencing of the event (trauma-related memories, nightmares, and flashbacks); (2) avoidant symptoms (trauma-related thoughts/feelings or external reminders); (3) a negative change in general responsiveness (about oneself or the world, negative affect, feelings of guilt or self-blame, social withdrawn, decreased interest in activities, and cognitive difficulties); and (4) increased arousal and reactivity (hypervigilance, irritability or aggression, attention and sleep disturbances, exaggerated alarm reaction) [4, 7].

On August 24, 2016, a Mw 6.0 earthquake hit Central Italy, overwhelming Amatrice and several minor towns and rural communities, with almost 300 deaths and leaving 30.000 people homeless. Reconstruction of the destroyed facilities was still lacking years after the earthquake. This situation hampered the recovery process even further and may having had further negative consequences on the population. The long-term psychological outcomes in the Center Italy population, however, are not yet known.

Prevalence rates of PTSD subsequent an earthquake are extremely heterogeneous across research and range from 4% to 67%; causes for this variability include the population considered, the age groups considered, the time elapsed since the event, the sample size, and the study design. It is worth observing here, that together with PTSD, depression is one of the most typical psychiatric disorders appearing in earthquake survivors several weeks or months after traumatic events. Moreover, both disorders tend to endure over time. In addition to the considerable mental health burden, research studies also indicate that PTSD and depression are related with improved risk of cardiovascular diseases, diabetes, functional impairment, lower quality of life, and mortality [8]. Clarifying the nature of the dysfunctional emotional in earthquake-exposed persons could allow choosing the most effective intervention approach allowing survivors to boost resilience and to acquire specific mental skills to manage threats [9].

The influence of natural disasters on mental health varies for several pre-, peri- and post-disaster factors [10]. Risk and vulnerability as well as disaster-related variables factors for the PTSD onset and its chronic course after seismic disasters include socio-demographic variables as well as disaster-related variables [2, 11, 12]. One of the first factors investigated is the loss of a family member or a loved one. The survivors who suffered a loss have a PTSD incidence of 39.10%, while those who have not experienced this event show a PTSD rate of 19.92%. In addition, being witnesses of death carries the PTSD’s risk developing for a 26.28%, against a 14.69% for those who did not witness the humans’ death [5, 13]. During a seismic event, it is possible to undergo slight or more serious injuries that may have disabling consequences [12]. Injuries cause a PTSD’s incidence of 23.28% against the 9.63% for the unharmed [5]. The risk of PTSD has constantly been proven to be associated with the severity of exposure to the disaster, with direct survivors at most risk [14]. There are numerous post-earthquake characteristics such as loss of houses that may enlarge the impact of the catastrophe for the ones involved. Many earthquake victims must adapt to living in temporary housing (tent camps, campers, hotels, or emergency housing solution), because their home has been destroyed or deemed not accessible [12]. Having suffered home damage involves a PTSD incidence of 38.49% against the 23.97 in absence of house damage [5]. Individuals who have underwent multiple movements are more at risk of PTSD developing [11]. Furthermore, the effect of socio-demographic variables such as gender, age, education, marital status, and occupation was explored [15–18]. The PTSD incidence in women was of 34.82% against the 22.57% for men [5]. Several studies reported that the young age represents a vulnerability source: children and adolescents have a higher risk of developing PTSD, sleep disorders, generalized and separation anxiety, panic attacks, substance abuse, and reactivity and cognition alterations [4, 19]. Moreover, for their troubles in the adaptation to new living conditions, older people seem to be at PTSD risk [20]. A further element is the education’s level: among individuals with low education, the PTSD incidence was of 31.56%, while for the upper level it decreased to 19.76% [5]. Finally, occupation seems a predictive variable in that unemployed people are more at risk of developing PTSD [11]. The extent to which the large-scale disaster leads to long-term consequences on mental health differ with these co-occurring factors, and thus awareness of these issues is critical to plan treatment intervention in the aftermaths of disasters. The persistence of PTSD symptoms encompasses medical and social costs, due to amplified comorbidities and functional impairments.

The main purpose of the current study was to estimate the long-term PTSD’s occurrence in a convenience sample from the Center Italy population. With this aim, we firstly obtained the Italian version of the first 16 items of part IV from the Harvard Trauma Questionnaire (HTQ) on non-exposed individuals exploring its psychometric properties [1, 21]. The HTQ is a cross-cultural screening instrument initially developed by the Harvard Program in Refugee Trauma to state trauma exposure and trauma-related symptoms in the Indochinese individuals living in America, and others exposed to potentially traumatizing experiences. Therefore, it is also relevant for use with non-refugees’ populations. The revised HTQ (HTQ-R) utilize the PTSD criteria from the DSM-IV version. This questionnaire was then adapted for other populations such as French, Arabic, Bosnian, Indian, Quechua and Tibetan [22–26]. Early identification of the symptoms, and/or prevention and intervention programs can reduce the long-term adverse outcomes. Early assistance or support, as well as professional treatment, may facilitate to prevent the onset of mental disorders or may reduce the gravity of mental illness should it develop. Moving from previous evidence on earthquake exposed individuals, we also aimed to explore the impact of the risk/vulnerability factors on PTSD, as measured by the HTQ, two-years after the Central Italy earthquake considering at the same time the viability of the four HTQ clusters for the clinic use [27]. We know of no other widely distributed Italian translation of the HTQ and, to our knowledge, no previous study has reported on the factor structure of the Italian version of the HTQ.

## Preliminary Study: Translation and Adaptation in Italian Language

The preliminary study consisted of translation to the Italian language and validation of the first 16 items of the HTQ IV part [21] investigating their psychometric properties. Specifically, we intended to replicate the findings of the validation accomplished in other nations, thus providing further evidence for the transcultural validity of this scale [23, 26, 28]. The primary goal of the HTQ is to identify individuals with traumatic disorders who might benefit from a treatment intervention. For our adaptation and translation, a four-step methodology was used [29] basing on the HPRT’s recommendations [30]. To ensure an accurate procedure for cross-cultural adaptation and linguistic validation, we have followed a translation/back-translation procedure. The preliminary step of the study consisted of the translation of the first 16 items of the HTQ IV part from English into Italian by two translators who were native Italian with high proficient in English and had practice of the topic. During the second step, the translators compared the forward versions with the original inventory and reconciled their differences. In the third stage, a native English professional translator not involved in the study performed the back-translation to guarantee accuracy and comparability. In the fourth period, an experts’ group (composed by two psychiatrists and three psychologists skilled in trauma field) discussed, adapted, and refined the terms of the questionnaire reaching a consensus for each sentence of the Italian version. Each item was read out aloud (with participants also following the reading of the text on paper-printed copies) and a group discussion followed, in which members were required to answer two questions: “What does this statement mean to you?” and “Is there any other terms that enables this connotation to be expressed more clearly?”). Answers yielded the final Italian version of HTQ, whose psychometric properties were then tested. Cronbach’s alpha for the HTQ in the present translation is reported in the results section. Data for this study was collected between May and November 2017 in Parma, Italy. To include a broader range of experiences, we used purposive heterogeneous sampling as well as convenience sampling strategies - for instance, referral from other participants.

## Methods and Measures

### Sample and Design

While a priori sample size determinations for AUC are highly susceptible to assumptions about the performance of the test, it was suggested that a sample size of 100 is generally sufficient to make a qualitative assessment of the utility of a test to run the factorial analysis [31, 32]. Given that the HTQ contains 16 statements, we estimated that for test validation we need ten subjects for each item. Thus, we aimed for a minimum sample size of 160 participants. The preliminary study received the approval from the Institutional Review Board (IRB) of the Parma University (Italy). A convenience sample was recruited from the students’ population attending the Medicine and Surgery Department of a University of North of Italy. Eligible subjects were invited by the research staff, trained in the study protocol and procedures by the principal investigator (the first author), for the study’s first occurred in May/June 2017. During this study, the 100% of the respondents (*N* = 254 in total) filled in the complete questionnaire without missing data. After six months (October/November 2017), they were invited to participate in the test-retest.

### Instruments and Assessments

The Harvard Trauma Questionnaire (HTQ) was created [1, 21] because the need for a cross-cultural tool to assess traumas and mass-violence consequences following of the gold standard Hopkins Symptom Checklist-25 (HSCL-25). The original study was carried out on 91 patients who received a clinical treatment [21, 23, 33]. It was initially conceived according to the Western perspective and the DSM-III-R diagnostic criteria (American Psychological Association, 1987). In its original format, the HTQ is a self-administered checklist consisting of four sections: 1) the first part containing 17 items about the exposure to different traumatic experiences (e.g.: life risks, lack of primary goods) on a four-points type-Likert scale (“No exposure”, “Indirect exposure”, “Witness”, “Protagonist of trauma”); 2) the second part consists of two open-ended questions about the traumatic experience; 3) the third section defines physical injuries (e.g.: beatings, suffocation), 4) the fourth part is a 30 items list with the first 16 questions investigating the PTSD, while the other points are specific additional elements for Indochinese refugees. All questions provide four response options: 1- “Not at all”, 2- “A little”, 3- “Quite a bit”, 4- “Extremely”. The test offers two findings: to establish the PTSD presence/absence, the procedure requires the sum of the first 16 items scores dividing it by 16. The threshold value is of 2.5 (raw score 40). Results of Mollica’s study showed that the internal consistency for the fourth part (0.96) was robust. Furthermore, the criterion validity analysis indicated, for a threshold of 2.5, a sensitivity of 0.78 and a specificity of 0.65 [21, 23]. Afterwards, Vindbjerg and colleagues [27] proposed the revised HTQ fourth part (HTQ-PTSD-R), where the 16 items were sub-divided according to the DSM-5 into the four PTSD clusters: a) Intrusion symptoms (items: 1, 2, 3, and 16); b) Avoidance (items: 11, 15); c) Negative alterations in cognitions and mood (item: 4, 5, 12, 13, and 14); d) Alterations in arousal and reactivity (item: 6, 7, 8, 9, and 10). This adaptation allows two advantages: a) a total score (the PTSD is present if the threshold value of 2.5 is exceeded); b) a raw score for each cluster. The Intrusion sub-scale could have a minimum score of 4 and a maximum of 16, the Avoidance sub-scale could have a minimum score of 2 and a maximum of 8, whereas the remaining clusters could have a minimum score of 5 till a maximum of 20. For the purposes of the present research, this modified version was used.

As concurrent instrument, the Screening Questionnaire for Disaster Mental Health (SQD) was utilized [34]. This is a valid self-report screening tool able to estimate the PTSD and depression occurrence following natural disasters. The SQD, firstly developed after Japan’s earthquake in 1995, was validated for the Italian after Aquila’s earthquake in 2013. It contains 12 statements with dichotomous answer (yes/no) expressed in a clear and spontaneous language, so that the questions are understandable for the elderly and people with an education’s low level. The items are classified into two sub-scales with a high discriminating efficacy: SQD-P for the diagnosis of probable PTSD (item: D3, D4, D6, D7, D8, D9, D10, D11, D12) and SQD-D for probable depression (item: D1, D2, D3, D5, D6, D10). The SQD-P sub-scale scores can arise three possibilities: zero-3 points “Slightly affected” (currently little possibility of PTSD); 4–5 points “Moderately affected”; 6–9 points “Severely affected” (possible PTSD). Instead, the SQD-D sub-scale scores can be evaluated only on two levels: 0–4 points “Less likely to be depression”; 5–6 “More likely to be depression”. Cronbach’s alpha mean value resulted of 0.86 for the SQD (0.79 for SQD-P and 0.76 for SQD-D, respectively). Concurrent validity, as measured by the Spearman correlation coefficient, was statistically significant for both tools: the correlation between the SQD-P with the Italian version of the golden standard tool Clinician*-*Administered PTSD Scale for DSM-IV (CAPS, [35]) was of 0.80, while the correlation between the SQD-D and the BDI-II was 0.76 [34]. The Binge Eating Scale (BES, [36]) was applied to explore the discriminant validity of the HTQ. BES is a self-report instrument that evaluates the behavioral and emotional/cognitive symptoms associated with the binge eating disorder. It comprised 16 items formulated on a four-point type-Likert scale. A value above the threshold of 27 identifies a probable eating disorder [37, 38]. The BES has been validated with the Structured Clinical Interview for DSM-IV Disorders (SCID) [39].

### Procedure

The preliminary study was promoted via University social media (forums, blogs, and social networks). Following the recruitment, a research assistant explained to the participants both the research aims and procedures asking their collaboration and signature of the informed consent. Participants filled the informed consent, the HTQ, the Screening Questionnaire for Disaster Mental Health (SQD) and the Binge Eating Scale (BES) completing all tests together with a questionnaire about socio-demographic data in a self-report mode. The criteria for exclusion from the study were: (1) history of severe brain injury; (2) diagnoses of intellectual disability or neurological damage; and (3) diagnoses of psychiatric disorders or being to drug intervention. Evaluations were administered during two periods (May/June 2017 and October/November 2017, respectively).

### Data Analysis

All analyses were carried out using the statistical program R version 4.0.1; lavaan [40] and sem [41] packages were used to perform the Confirmatory Factorial Analysis. Test-retest reliability refers to temporal stability and can be operationalized as a correlation of scores of a test that has been administered at different time-points [42]. It was assessed through the Pearson’s correlation coefficient on step 1 and step 2 scores. The convergent and construct validity was explored examining the association with the SQD-P scores through the Pearson’s coefficient test. The discriminant or divergent validity coefficient was calculated through the association with the BES, given that eating disorders and PTSD are very distinct concepts. Furthermore, in order to verify if and to what extent the 16 items of the HTQ agree with the partition into the DSM-5 four clusters, the structural validity of the reference model [27] was explored through a Confirmatory Factorial Analysis (CFA): loadings (correlation coefficients between the items and the identified component) and model indexes of fit (Root Mean Square Error of Approximation - RMSEA, Standardized Root Mean Square Residual - SRMR, Tucker - Lewis Index - TLI, Comparative Fit Index - CFI) are reported.

The internal consistency, which refers to the scale reliability or the degree of consistency among the scores on items of an instrument concerning a particular sample or subsample of a population [42] was determined by computing the Cronbach’s alpha (Cronbach 1951). Calculating Cronbach’s alpha is particularly useful investigating the reliability properties of an instrument that does not have right or wrong answers [43], such as the HTQ-symptom scale. Cronbach’s alpha value above 0.5–0.7 was generally deemed to suggest sufficient reliability for a tool to be applied to make group comparisons [44–47]; instrument with coefficients above 0.85 are considered reliable enough for individual comparisons. As regards the internal consistency, the interitem and the corrected item-total correlation coefficients for the HTQ’s items were also computed using Cronbach’s alpha. The coherence between every single item and the construct, as operationalized by the total score, was estimated basing on the corrected item-total correlation coefficients calculating the correlation between each individual item and the total scale with the item of interested eliminated. Data screening was performed to check for accuracy of data entry and to check for parametric test assumptions. Unless stated otherwise, the criterion for statistical significance was set at alpha = 0.05.

**Fig. 1:**
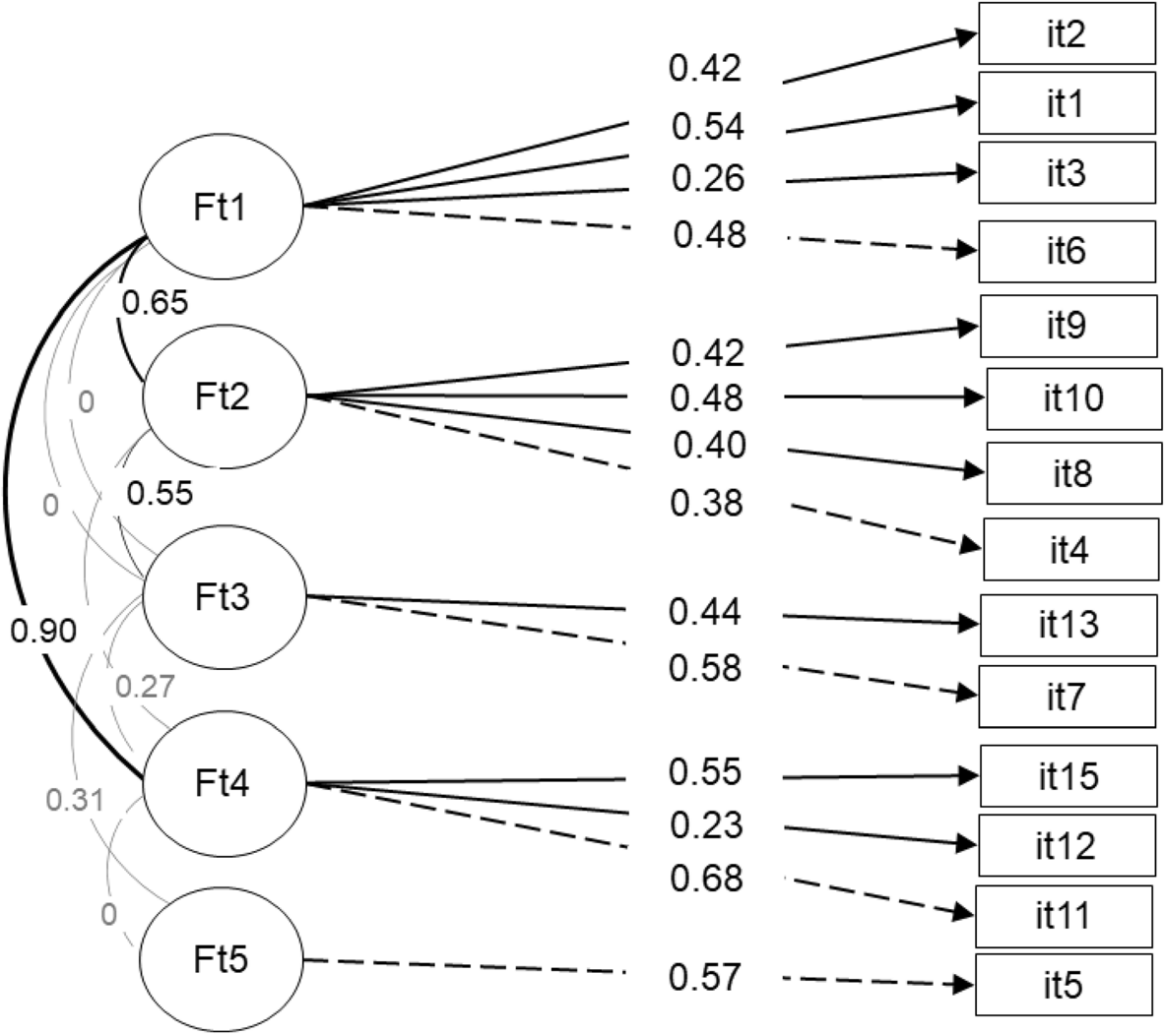
Latent variables emerging from Confirmatory Factorial Analysis

## Results

### Sample Characteristics

For the first study two hundred fifty-three subjects were recruited. Of those, 21 participants obtained an SQD-*P* ≥ 6 score, thus resulting above the cut-off for a diagnosis of probable PTSD and were therefore excluded from the following analyses. The remaining 232 participants (62.5% females, 22.1 ± 2.1 years old) were mainly resident in the province of Parma (Italy; 79.4%). One hundred fifty of them (64.7%, M = 22.1 years, SD = 2.1) agreed to be called back for the re-test. Each subject provided consent, and potential participants were excluded if they were unable to provide written informed consent.

### Test Scores

Scores of the all tests were summed to provide an overall score for the HTQ with M = 1.505 (SD = 0.37, range 0–2.75) for the test phase, and with M = 1.522 (SD = 0.35, range = 1–2.7) for the re-test, respectively. The mean score is very far from 2.5 that represent the threshold value, confirming the absence of a post-traumatic symptomatology. Scores on SQD were also summed to provide an overall score for both psychological distress (M = 1.99, SD = 1.71, range = 0–5) and depression (M = 1.29, SD = 1.21, range = 0–5).

### Model Fit, Internal Consistency, Convergent and Divergent Validity

In Fig. 1 the latent variables emerging from Confirmatory Factorial Analysis are reported. To verify the structure of the underlying latent constructs, a confirmatory factor analysis (Maximum Likelihood method, Varimax rotation with Kaiser normalization; KMO = .833, Bartlett’s χ^2^_120_ = 1043.57, p.000) was run: it showed that a five-factors solution was the best model, with satisfactory indexes of fit (RMSEA = 0.055, p. 0.260; CFI = .927; TLI = .908; SRMR = 0.051).

Intrusion symptoms were saturated on Factor 1(items: 1 “Intrusive recollection”; 16 “Psychological and physiological distress”; 2 “Event recurring”; 3 “Recurrent dreams”), alteration in arousal and reactivity on Factor 2 (items: 6 “Exaggerated startled response”; 10 “Irritability or anger”; 9 “Hyper-vigilance”; 8 “Sleeping difficulty”; 4 “Feeling of detachment from others”; 14 “Sense of foreshortened future”), negative alterations in cognition and mood on Factor 3 (items: 7 “Difficulty concentrating”; 13 “Diminished interest in activities”), and avoidance strategies on Factor 4 (items: 11 “Effort to avoid activities”; 15 “Effort to avoid thoughts”; 12 “Memory impairment”). A single item (5 “Inability to feel emotions” saturated on factor 5). This statement was frequently misunderstood, exhibiting a low endorsement and loading in other validation studies (see [24, 27]).

The convergent and divergent validity of the HTQ was explored by calculating its Pearson correlations with well-established measures of PTSD (SQD-P subscale) and depression (SQD-D sub-scale), and binge eating disorder (BES), respectively. HTQ held a fair positive correlation with the SQD-P subscale (r = 0.65, *p* < 0.05) and a significant positive correlation with depression, as measured by the SQD-D (r = 0.47, p < 0.05). Conversely, the BES showed a very weak correlation with HTQ (r = 0.25, p < 0.05).

The internal homogeneity was calculated using Cronbach’s alpha, and test-retest reliability was evaluated through the Pearson’s correlation. Cronbach’s alpha is the measure of the reliability and consistency of the sampling instrument and examine whether all the data were measuring the same underlying construct, because the various components were intended to represent specific, correlated aspects of one global construct rather than separate constructs [44]. Data analysis showed that HTQ had a good internal consistency with a standardized alpha of 0.827 (95% CI 0.819–0.835) for the test phase and 0.80 for the re-test phase, respectively.

Finally, the inter-item correlation between test and re-test was calculated with the Pearson’s Product Moment Correlation coefficient on the total HTQ score suggesting a very good temporal reliability or stability, t (161) = 14.069 with r = 0.743 (*p* value <0.05), if we refer to the lower thresholds typically referenced by health professionals [48].

## Study on PTSD Assessment among Earthquake Survivors

This part of the study aimed to replicate and extend prior research on the psychometric properties of the HTQ in an Italian sample from the population exposed to the earthquake of 2016 held in the Central Italy, which resulted in great psychological distress for the inhabitants, who had not yet recovered from the psychological, social, and economic consequences. The data collected in this part of the study served to estimate the PTSD long-term occurrence following seismic events investigating the risk and vulnerability factors for the PTSD development and to refine the data collected in the preliminary study. The traumatic event was on 24 August 2016 at 3.36 am in the Valle del Tronto, when an earthquake of 6.0 magnitude caused 299 deaths, 238 survivors were extracted from the rubble and hundreds of injured in the district of Accumoli (RI), Amatrice (RI), and Arquata del Tronto (AP). The main earthquake was followed by thousands of aftershocks in the subsequent months with a severe psychological burden on the whole population. The great damage to buildings and the inability of entire countries have produced a large amount inhabitant from the districts of Ascoli Piceno, Macerata and Rieti being forced to leave their homes. In the later months, the earthquake swarm hit 140 municipalities involving Abruzzo, Lazio, Marche, and Umbria. All residents of these areas have been directly exposed to the disaster, with several individual experiences (mourning, loss of housing, work disruption, media impact). From these survivors’ population we recruited a sample for the participation at the second phase of the study. Specifically, with the following purposes: (i) establishing the HTQ and SQD convergent validity through significant positive correlation between measures assessing the same or similar constructs (SQD-P and SQD-D). In detail, the correlation between instrument and the HTQ is expected to be significant but less when compared to the correlation between the HTQ with the SQD-D. (ii) Because of the purpose of testing is to identify individuals with PTSD, increasing the identification accuracy through a comparison with the normative sample drawn from the general population. The primary evidence needed to support this diagnostic decision is test specificity and sensitivity. Specificity is the correct classification of typically individuals as having no psychological disorders. Sensitivity refers to the correct classification of individuals with PTSD as having PTSD. We address the criterion validity issue, calculating the ROC curve, which will reveal an optimal HTQ’s cutoff. (iii) In the effort of assessing the prevalence of PTSD among adult survivors, we predicted a worse traumatic symptomatology for individuals from the area closest to the epicenter (Red zone). Yet, no studies so far have focused on measuring how exposure to earthquake for populations in the Centre of Italy may have influenced their survivorship. This investigation on the post-traumatic responses and its association with risk/protection factors responds therefore to the call for studies of long-term outcomes of potentially traumatic events.

## Methods and Measures

### Ethic Approval

This study was firstly approved by the Institutional Review Board (IRB, Prot. 0082/2017) of the University of Parma (Italy) and then it received the approval by the Ethical Committee of the Marche Region (CERM, Prot. 2018 24). A written consent form was used to communicate to the eligible individuals the topic and purpose of the research, the voluntary and unpaid nature of participation, the possible side effects (unpleasant memories), and the measures taken to protect confidentiality and anonymity. All data were analyzed anonymously.

### Sampling Procedures, Participants and Setting

The minimum sample size was estimated considering the expected proportion P1 = .25 according to the available literature data [5], a maximum marginal error of the estimate d = .05 (d < ¼ P1, as recommended by [49]), for a 95% CI as in the following equation:

N = [z × α/2 × P1(1-P1)]/d = (1.962 × .25 × .75)/.052 = 288.12. The result required a total of *N* = 288 individuals.

Participants for this study were recruited through contacts with the municipalities, by newspaper and online advertisements and notices requesting to provide contact information so that the research staff can reach them to review eligibility and discuss next step. The study used flyers and oral presentations at various sites to recruit participants; these sites included collaborating organizations, and diverse associations representing the earthquake’s victims of the. In three rural villages, the researcher first obtained permission from the local authorities, and then posted flyers. During a phone screen, study staff confirmed potential interest, eligibility answering any question the participant may have about the study. After checking the inclusion and exclusion criteria, the eligible subjects were scheduled for a consent visit for additional screening and confirm eligibility. The inclusion criteria were: 1) age (over 18 years); 2) possible use of drugs for the clinical disorders treatment (benzodiazepines, antidepressants or other); 3) written informed consent; 4) residence and being during the earthquake of 2016 in one of the municipalities of Ascoli Piceno (Acquasanta Terme, Appignano del Tronto, Ascoli Piceno, Arquata del Tronto, Cossignano, Force, Maltignano, Offida, Roccafluvione, Venarotta), Macerata (Caldarola, Treia, Tolentino, Ussita, Visso), and Rieti (Accumoli, Amatrice). The exclusion criteria were: 1) presence of neurodegenerative diseases (including Parkinson’s, Alzheimer’s, and Huntington’s disease, amyotrophic lateral sclerosis); 2) occurrence of neuro-developmental disorders (e.g.: autism spectrum disorder or intellectual disability); 3) participation in other clinical trial. Three hundred and ten potential participants were contacted and offered study participation; fifteen of them did not meet inclusion criteria, and seven declined to participate because of conflict with their work schedule. Thus, the sample of the present study was initially composed of 288 individuals. Written informed consent was obtained from all participants before performing any study procedures.

### Instruments and Assessments

To obtain more knowledge on the influence of earthquake on the individuals, we decided to include an additional questionnaire on the socio-demographic and earthquake-related factors, as well as a widely used assessment tool for PTSD symptoms. Questionnaire was constructed for the current research to assess exposures during the earthquakes, negative experiences from the earthquake and the impact of the earthquakes on the life. The questionnaire contained dichotomous or multiple-choice answers. The statements collected data on participant’s gender, year of birth, place of residence, educational qualification, marital status, profession, delocalization in the post-earthquake period, current housing situation, damage to houses, psychological diagnoses, current psychotherapy or pharmacological treatments, and life events suffered in the last year. The incorporated traumatic experiences during the earthquakes were seeing someone who were injured or killed, whether they assisted in rescue efforts and if family members (relatives or close family) were killed, injured, or trapped. The statements were based on previous traumatic exposure instruments and on consultations in the team of investigators with experience from the research field. Post-Traumatic Stress Related Symptoms (HTQ-PTSD-R) were measured by the Harvard Trauma Questionnaire (HTQ) Part IV [1, 21] and the SQD [34], already described in the first part of the study. The study used the HTQ adapted Italian version obtained from the preliminary research. Given that the HTQ is considered a gold standard measure with established reliability and validity in association with distal events, and provide the most conservative estimate, the HTQ guidelines were used.

### Procedure

A psychology graduate (the second author) was trained at the Cognitive Psychology Laboratory in the Department of Medicine and Surgery of the University of Parma (Italy) to manage the HTQ and SQD tools with the earthquake victims. She administered the socio-demographic questionnaire (see Instruments and assessment paragraph) in a face-to-face interview to enrolled participants in the districts belonging Ascoli Piceno, Macerata, and Rieti. Data was collected from June and October 2018 approximately 20 months after the earthquakes within the frame of specific environments to protect the privacy of the participant (home administration or in structured contexts). After a verbal consent, each subject filled in the written informed consent. Afterwards, they responded to the socio-demographic interview, the HTQ, and the SQD. Each administration took approximately 10–30 min for completion and was held at the participants’ home or at a place where they felt comfortable as long as confidentiality was assured. Prior to the interview the researchers explained that it was likely that the socio-demographic and questionnaires’ affirmations would recall memories related to the events; therefore, participants could experience some discomfort or distress as a result. The information contained on the subjects’ information booklet was then explained and clarified. The participants were reminded that their information would be handled in agreement with ethical guidelines and confidential procedures, and that they could withdraw their collaboration at any time during or after the interview. The involved trial has been conducted in accordance with the Helsinki Declaration.

### Data Analysis

All analyses were carried out using the statistical program R version 4.0.1; ROCit [50] and MuMin pakages [51] were used for ROC curve analysis and generalized linear models’ selection, respectively. The data of the clinical sample served to evaluate the HTQ sensitivity, the PTSD occurrence at two years after the earthquake and the influence of risk and vulnerability factors on post-traumatic responses. We calculated the optimal cut-off points for the scales using a ROC curve analysis, considering the SQD-P cut-off >6 as golden standard. The Area Under Curve (AUC) is treated as a synthetic goodness of fit measure indicating how well a given predictor can separate classes of a dependent variable. Sensitivity and specificity were investigated for several cut-offs, following the guidelines of Zweig and Campbell [52].

Data obtained with individuals recruited from survivors were compared to those of the normative sample (one sample t-test). The sensitivity was calculated through the associations among the PTSD cases identified from the reference test (SQD-P) and those suggested from the HTQ test (chi - squared test). The impact of different socio-demographic and earthquake-related variables on HTQ scores was explored through backward stepwise logistic regressions to get the best predictors for PTSD. With the purpose to investigate the relationship between traumatic symptomatology and earthquake-related factors, during data processing, subjects were distributed according to the area of origin into three subgroups: 1) Red zone, where participants suffered additional traumatic experiences (mourning, exposure to deaths, destroyed zone such as in Amatrice or Arquata del Tronto); 2) Orange zone, with participants coming from the district of Macerata; 3) Yellow zone, whose members came from the Ascoli Piceno district.

## Results

The HTQ-PTSD-R average score of the sample exposed to the earthquake, obtained from raw scores following the guidelines of Mollica et al. [30], (M = 1.92, SD = 0.61) was compared with the normative population mean score found in the preliminary study (M = 1.505, SD = 0.37), showing a significant, discrete difference (one sample *t*-test: t_280_ = 14.406, *p* < .001; Cohen’s d = .681).

### Demographic Characteristics of respondent’s in Study Variables

Table 1 shows the socio-demographic and earthquake-related characteristics of the sample. Finally, the study involved 281 participants because nine of them dropped during the test. All individuals have directly suffered or witnessed multiple traumatic events. There was a prevalence of female gender (160 women, 56.9% of the sample) and a mean age of 51.2 years (SD = 17.5, range = 18–90). Red zone included 89 individuals, while Orange and Yellow zone 103 and 89 individuals, respectively. Most participants presented a high level of education (159 of the respondents, 56.6%), are married (145 of the respondents, 51.6%), and in part they have lost their work (50 of the respondents, 17.8%). A 24.6% of survivors experienced mourning and only 5.7% suffered injuries. Moreover, 9.9% reported being exposed to death. Most of the sample (62.6%) reported that their own home was damaged requiring full repair. During the 2018, only 39.5% of the sample lived in own home whereas the 30.9% is settled in SAE. Anxiety/panic attack and PTSD diagnosis were reported by 5.7% and 3.9% of survivors, respectively. An 88.6% of participants declared that it was never clinically evaluated (Table 1).

**Table 1:** Socio-demographic characteristics of participants in the second study: N (percentages)

Participants characteristics (*N* = 281)	N (%)
Zone	Red zone^1^: 89 (31.7); Orange zone^2^: 103 (36.7); Yellow zone^3^: 89 (31.6)
Gender	Females: 160 (56.9); Males: 121 (43.1)
Education^4^	High level: 159 (55.6); Low level: 122 (43.4)
Marital status	Married: 145 (51.6); Unmarried: 76 (27.1); Widower: 27 (9.6); Separated/Divorced: 17 (6.1); Cohabitant; 16 (5.8).
Work	Not Working^5^: 142 (50.5); Working: 139 (49.5)
Loss of Work	Yes: 50 (17.8)
*Work resumption*	*Same occupation: 19 (38.0); New occupation: 14 (28.0); No occupation: 17 (14.0)*
Mourning	None: 212 (75.4); Yes, experienced^6^: 69 (24.6)
Injuries	None: 265 (94.3); Yes, experienced^7^: 16 (5.7)
Exposure to death	Nobody: 253 (90.1); Yes, experienced^8^: 28 (9.9)
Damaged home^*^	Damage: 176 (62.6); No damage: 105 (37.4)
House condition during the year following the earthquake	Unusable for structural damage: 142 (50.5); Suitable: 105 (37.4); Partially accessible: 13 (4.6); Temporarily unusable: 12 (4.3); Unusable for external risk: 9 (3.2)
Relocation^*^	More displacements: 90 (32.1); Own home: 87 (30.9); Different house: 67 (23.8); Precarious accommodation: 14 (4.9); Hotel: 10 (3.6); Other (e.g., camper, container): 13 (4.6)
Habitation during the year following the earthquake	Own Home: 111 (39.5); SAE: 87 (30.9); Different House: 66 (23.5); Hotel: 14 (4.9); Other (e.g., camper, container): 3 (1.1)
Other events	No: 209 (74.4); Yes^9^: 72 (25.6)
PTSD diagnosis^*^	No evaluation for PTSD: 266 (94.7); Positive: 11 (3.9); Negative: 4 (1.4)
Other psychiatric diagnosis	Never evaluated for other mental disorders: 249 (88.6); Anxiety / panic attacks: 16 (5.6); Insomnia: 8 (2.8); Depression: 8 (2.8)
*Treatments*	*Pharmacological: 14 (4.9); Psychotherapy; 6 (2.1)*

^1^*Accumoli (1, .4%), Acquasanta Terme (21, 7.5%), Amatrice (14, 5%), Arquata del Tronto (46, 16.4%), Maltignano (7, 2.5%)*

^2^*Caldarola (32, 11.4%), Tolentino (6, 2.1%), Treia (26, 9.2%), Ussita (14, 5%), Visso (25), 8.9%)*

^3^*Appignano del Tronto (26, 9.2%), Ascoli Piceno (15, 5.3%), Cossignano (10, 3.5%), Force (9,3 .2%), Offida (10, 3.6%), Roccafluvione (12, 4.3%), Venarotta (7, 2.5%)*

^4^*High level: College degree, postgraduate; Low level: Primary and High School*

^5^*Unemployed: 61 (21.7)%; Retired: 66 (23.5)%; Student: 15 (5.3%)*

^6^*Friends: 56 (19.9); Relatives: 16 (5.7)*

^7^*Minor injuries: 11 (3.9); Serious injuries: 3 (1.1); disability: 2 (0.08)*

^8^*Acoustics: 6 (2.1); Friends: 9 (3.2); Relatives: 1 (.04); Unknown:1 (.04)*

^9^*Mourning: 32 (11.4); Disease: 21 (7.4); Smash-up: 5 (1.8); Mugging: 3 (1.1); Others: 13 (4.6)*

^*^Caused by the earthquake

### Convergent Validity

**Fig. 2:**
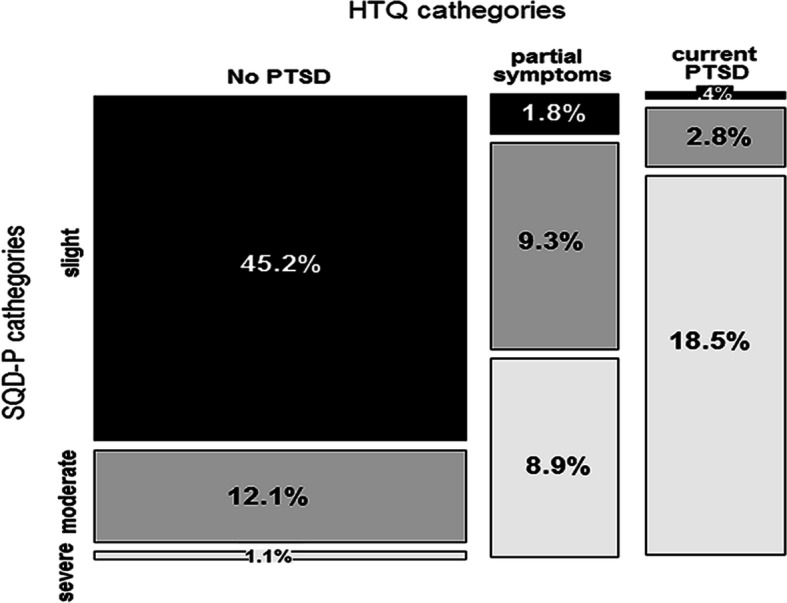
Prevalence of individuals exposed to the earthquake in 2016 diagnosed with probable PTSD, partial PTSD or without PTSD according HTQ and SQD, respectively

In this phase of the study, the average scores for HTQ and SQD-P sub-scale were 1.92 ± .61 (range = 1–3.80) and 3.93 ± 2.5 (range = 0–9), respectively. The SQD-D mean was 2.37 ± 1.8 (range = 0–6), and the 17.44% of the sample (*N* = 49) was assessed as probably depressed. As seen in Fig. 2, using the original cutoff of 2.5 - as suggested by Mollica et al. [30] developers of the scale - the 21.7% (*N* = 61) of the sample is considered symptomatic of PTSD, followed by 19.9% who reported a partial PTSD (*N* = 56). Meanwhile, according to the SQD-P subscale the 28.5% (*N* = 80) of the sample showed a probable PTSD, and the 24.2% (*N* = 68) a partial PTSD. The diagnostic categories of SCD-P and HTQ emerged as associate (χ^2^_4_ = 206.21, *p* < .001, Sakoda’s Adjusted Contingency Coefficient C_adj_ = .797), mainly the absence of PTSD (standardized adjusted cell residuals: z = 11.97) and its positive occurrence (z = 11.1),

The zero-order correlation between HTQ total score and SQD-P subscale was positive and very good (Pearson’ r = .849, *p* < .001, 95% IC: 0.814–0.88879), as well as the zero-order correlation between HTQ total score and SQD-D (r = .706, *p* < 0.001, 95% IC: 0.642–0.76)). However, despite the partial correlation HTQ-SQD-P, controlled for SQD-D, remained quite strong and significant (r = .669, *p* < .01), the partial correlation HTQ-SQD-D, controlled for SQD-P, disappeared (r = .064, p .279).

The SQD-P scale was strongly related to the Intrusion (r = .752, *p* < .001, 95% CI: .696–.798) and Hypervigilance (r = .824, p < .001, 95% CI: .783–.859) scales, and rather well to Avoidance (r = .598, p < .001, 95% CI: .517–.669) and Negative mood (r = .587, p < .001, 95% CI: .505–.659).

### PTSD Status. Optimal Cut-Offs, Specificity, and Sensitivity

**Fig. 3:**
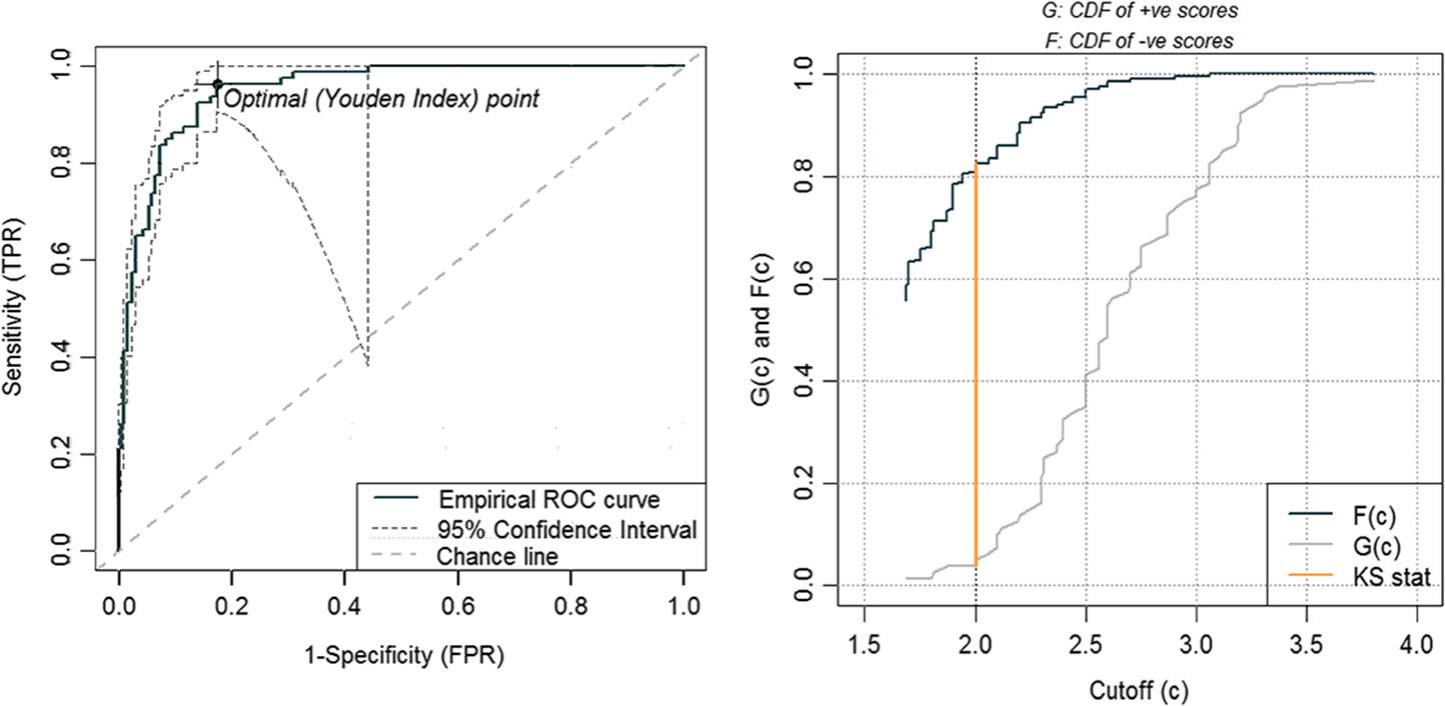
ROC curve and optimal cut-off point

To assess whether an alternative HTQ cut-off provided a better threshold for identifying PTSD, we calculated the Area Under the Curve (AUC) in a ROC analysis (Fig. 3) for the prediction of slight-to-severe post-traumatic symptoms, as measured with the HTQ, and to detect the optimal cut-off points. The AUC was = 0.951 (95% CI: .917–985), indicating a very good separation ability. We considered a score = 2 as optimal cut off to discriminate between people who had moderate-to-severe symptoms and those with milder or no symptoms, according to the convergent indications of the Youden index (0.773) and the Kolmogorov – Smirnov (K-S) statistics: sensibility was = 0.963 and specificity = 0.189. Tremblay et al. [53] have already suggested a cut-off of 2.0 for positive cases based on PTSD-R (Table 2).

**Table 2:** Model selection table of PTSD predictors: backward logistic regressions

Model	b_0_	b_1_						df	-2LL	AICc	Delta	Weight
		Zone	Age	Gender	Damages	Mourns	Time					
Gender + Age	1.29		−0.024	+				3	−179.129	364.3	0.00	0.389
Time	1.25						+	5	−177.141	364.5	0.16	0.359
Zone	−0.44	+						3	−179.668	365.4	1.08	0.227
Damages + mourn	0.32			+	+			3	−181.868	369.8	5.48	0.025

### Association between respondent’s Characteristics, Earthquake-Related Factors, and PTSD Status

**Fig. 4:**
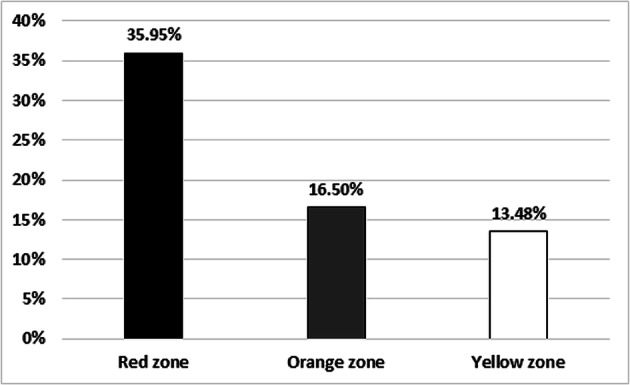
Impact of PTSD (according to the HTQ cut-off) among the recruitment sites

Regardless of the zone, among the socio-demographic variables (gender, age, educational level, marital status, and profession), only gender and age significantly predicted PTSD (backward logistic regression: LRT_χ2_ = 21.98, *p* < .001, Hosmer-Lemeshow pseudo-R^2^ R^2^_H-L_ = 0.059). Women appeared more exposed to the risk than men (OR = 2.17, 95% CI 1.317–3.642), while younger people were more secure than older ones (OR = 0.97, 95% CI 0.961–0.991). Proximity to epicentre significantly predicted the PTSD onset (LRT_χ2_ = 20.9, *p* < .001, R^2^_H-L_ = 0.055; see Fig. 4): The Red zone inhabitants were over three times more likely to experience PTSD symptoms than people living in the Orange (OR = 3.27, 95% CI 1.821–5.981) as well as in Yellow zone (OR = 3.36, 95% CI 1.828–6.299).

Among the factors contemporary or immediately following the earthquake (home damages, bereavement, personal injury, exposure to death risk), home damage and bereavement were predictive of PTSD symptoms (backward logistic regression: LRT_χ2_ = 16.5, p < .001, R^2^_H-L_ = 0.043): the likelihood of developing symptoms was more than twice in people whose homes had been damaged (OR = 1.92; 95% CI 1.121–3.329), while it was halved in those who did not suffer from bereavement (OR = .494, 95% CI 0.274–0.882).

Among the earthquake long-term consequences (job loss, relocation and time spent away from home, categorized as “never”, “<3 months”, “3–6 months”, “6–12 months”,” > 12 months”), the job loss was analysed separately, because it only applied to 151 participants (130 subjects were retirees or pre-earthquake unemployed). Analysis indicated that when participants did not lose their jobs, there was less probability to evolve PTSD (OR = 0.384, 95% CI 0.189–0.779; LRT_χ_ = 7.1, *p* < .01, R^2^H-L = 0.037). The time spent away from home (but not the type of the relocation) had a significant effect (LRT_χ2_ = 25.96, *p* < .0001, R^2^_H-L_ = 0.068): the likelihood of developing PTSD was significantly lower in people who could remain at home than in those who were taken away for 6–12 months (OR = .106, 95% CI 0.037–0.279) or more than a year (OR = .331, 95% CI 0.175–0.602), while it was analogous to the probability of who has been away for 3–6 months (OR = .345, 95% CI 0.094–1.314) and less than three months (OR = .445, .95% CI 0.178–1.125).

The model selection (Akaike’s information criterion corrected, AICc; see Table 2) identified as best predictors of PTSD symptoms the participants’ gender and age (Akaike’s weight *w* = 0.389) and the time spent away from home (*w* = 0.359).

In conclusion, the Cronbach alpha of the Italian version of the Harvard Trauma Questionnaire [1, 21] (a = 0.83 for the test and a = 0.80 for the re-test) revealed a good internal consistency. These scores fall within the range (0.80 ≤ α ≤ 0.96) seen in the literature, suggesting that the items were well validated and adequately understood by participants. Results on the convergent and discriminant validity of the HTQ and the SQD and BES were also satisfactory. The correlations between both the first measures were significant and in the expected directions. The clinical sample exposed to a potentially traumatic confirmed its eligibility (M sample = 1.92; M population = 1.53; t (83) = 6.716; *p* value <0.001). The correlation between HTQ and SQD-P was higher, with a great index result (r = 0.85). The HTQ showed a convergent validity like that of SQD-P subscale. Furthermore, findings suggested a good correlation between HTQ and the SQD-D subscale (r = 0.71), due to the HTQ items 4, 5, 12, 13 and 14 that addressed negatives mood and cognitions.

## General Discussion

The primary aim was to assess the prevalence of PTSD among the adult survivors of the earthquake of 24 August 2016. The issue of psychiatric morbidity after the earthquake needs to be explored to promote positive mental health among the earthquake victims. An important finding of the present investigation is that two years after a devastating earthquake, PTSD is prevalent among the 21.71% of the adult survivors and that this value does not differ so much from the mean percentage of 23.66% reported by the literature [5]. This suggests the properties of the HTQ as a scientific tool useful for professionals since it provides a diagnosis of PTSD in which the severity level is specified according to the DSM-5 criteria [27].

In detail, our data indicated a higher PTSD prevalence in the red zone compared to the other two zones, an outcome firstly anticipated, since the red zone was the epicenter and suffered extensive damage, loss of life and misplacement of entire municipalities. Our study confirmed the proximity to the epicenter as a risk factor [14]; being the protagonist of a natural catastrophe emphasizes feelings of powerlessness, vulnerability and panic experienced in such traumatic situations [54]. Thus, PTSD symptoms are suffered by the survivors even almost two years after the disaster requiring precise diagnosis and intervention to prevent long-term psychiatric morbidity. The study findings highlight that PTSD was significantly associated with gender and this discovery agrees with previous studies conducted abroad [4, 5], and in Italy [15, 16, 55, 56]. This female vulnerability can be attributed to anatomical features (e.g., sexually dysmorphic insula development), genetic susceptibility (greater PTSD inheritance), and by cultural variables (such as greater emotional attachment and dependence to the family) [4, 57]. Conversely, men seem less prone to PTSD [14, 19, 58]. Furthermore, earthquake consequences such as mourning, injuries, death exposure and home damage are confirmed as risk factors. Despite marital status, age and delocalization were considered vulnerability variable [11, 17, 56, 59], our study does not provided evidence in this regard. The presence of PTSD was small among the survivors with higher education levels. Higher educational level enhances the individual’s trauma understanding ability, which could improve confidence in physical as well as mental health, and use of functional coping strategies [18, 20, 60]. Previous research suggested that lack occupation is another risk factor for PTSD development [11], and our findings indicated higher rates of PTSD for unemployed and participants who lost their job.

An earthquake has serious concerns on the quality of life for people directly exposed to the disaster and there are consequences that can increase the PTSD development and its chronic course. The consequences of the earthquake affected much more the survivors living near the epicenter revealing similar findings to previous investigations [14]. Our study confirmed that deaths, damage to health, loss of home and exposure to deaths are risk factors [5, 13].

## Limitations

Our study is not without limitations. The aims of this research were to assess the long-lasting post-traumatic responses of exposure to earthquake of different districts during 18 weeks of data collection period in 2018, thus it may not represent the whole survivors of earthquake in Central Italy. The sample size is rather modest. Study results recommend executing further larger sample in other settings for better generalization of the findings. Another shortcoming is that the distribution of the gender was not homogeneous, as women were more frequently represented in the sample. The administration of the instruments took place about two years from the earthquake, and the impact was revealed dissimilar according to the area: some countries restarted normal activities while other areas were still facing the management of the population in the SAE. A further limitation is not having explored the influence of social or family support, since it could affect personal resources in managing traumatic events. Additional drawback was that the inter-rater reliability of diagnoses could not be investigated. Therefore, it remains unclear to what extent these symptoms would correlate with formal clinical diagnoses based on the DSM or ICD. This had implications, as the sensitivity to change could not be examined against diagnoses based on structured clinical interviews. Hence, the possibility that the HTQ poses some bias in diagnosis should not be ignored, and the combination of self-reports and clinical-based criteria should be addressed in future. Finally, both Cronbach’s alpha values and the lack of correspondence between the previous revision [27] and our data toward some items from the first 16 included in the IV part of the HTQ, suggested the need of revision or addition of extra items. Last revised version (HTQ-5) implemented to measure the trauma-related symptoms according to the new DSM-5 diagnostic criteria for PTSD [61] and the addition of the extra items has not changed the mean score of 2.5 as a provisional basis to be considered as checklist positive for PTSD, but authors recommended using the 2.5 cut-off with caution. Our research, as other authors [53] have suggested the best cut-off of 2.0 in discriminating for PTSD positive cases.

## Conclusions

The current study adds to the growing literature supporting the validity of the HTQ as screening tools of PTSD, even if it would be better an integration with the additional items included in the HTQ-5 [61]. An updated Italian version could offer large potential benefits to public health services, since other tools like the PCL-5 for screening the DSM-5 symptoms of PTSD, making a provisional PTSD diagnosis or monitoring symptom change during and after treatment there is no Italian validation.

First, the HTQ is an easily administered screening tool with adequate psychometric properties, allowing practitioners to distinguish individuals who revealed trauma symptomatology. When a high number of victims are involved, it is essential to have fast screening questionnaires that identify possible pathologies addressing the suffering individuals to appropriate therapeutic pathways. In addition, the first items of the HTQ IV part is a useful instrument in the clinical or research field, specifying a severity score for each cluster, more than reporting the presence/absence of PTSD. This questionnaire does not require enormous healthcare costs and it is different from other more invasive tools. Overall, the HTQ can be useful in catastrophes with a high number of victims. The benefit of such a list would be a shorter time of administration that, in turn, would increase the number of individuals having access to professional care. Additionally, this investigation provides further evidence for the value of the scale in a new country, namely, Italy. In conclusion, the PTSD prevalence of 21.71% after two years from the earthquake should encourage Italian institutions to plan psychological intervention not only in the emergency period, but also for the population reintegration.

## Data Availability

All data generated and analyzed during the present study are not publicly available for the protection of privacy right of participants.

## Abbreviations

BES: Binge Eating Scale
CERM: Comitato Etico Regione Marche
DSM: Diagnostic and Statistical Manual of Mental Disorders
GAD: Generalized Anxiety Disorder
HSCL: Hopkins Symptom Checklist
HTQ: Harvard Trauma Questionnaire
IRB: Institutional Review Board
MDE: Major Depressive Episode
OCD: Obsessive-Compulsive Disorder
PTSD: Post-traumatic Stress Disorder
SQD: Screening Questionnaire for Disaster Mental Health
SAE: Emergency Home Solution

## References

[1] Mollica R, Caspi-Yavin Y, Bollini P, Truong T, Tor S, Lavelle J. The Harvard trauma questionnaire. Validation a cross-cultural instrument for measuring torture, trauma, and posttraumatic stress disorder in Indochinese refugees. J Nerv Ment Des. 1992;180:111–6. 1737972

[2] Bianchini V, Giusti L, Salza A, Cofini V, Cifone MG, Casacchia M, Fabiani L, Roncone R (2017). Moderate depression promotes posttraumatic growth (Ptg): a young population survey 2 years after the 2009 l’Aquila earthquake. Clin Pract Epidemiol Ment Health.

[3] Dell’osso L, Stratta P, Conversano C, Massimetti E, Akiskal KK, Akiskal HS (2014). Lifetime mania is related to post-traumatic stress symptoms in high school students exposed to the 2009 L’Aquila earthquake. Compr Psychiatry.

[4] Farooqui M, Quadri SA, Suriya SS, Khan MA, Ovais M, Sohail Z, et al. Post traumatic stress disorder: a serious post-earthquake complication. Trends in Psychiatry Psychother. 2017;39(2):135–43. 10.1590/2237-6089-2016-0029. 10.1590/2237-6089-2016-0029 28700042

[5] Dai W, Chen L, Lai Z, Li Y, Wang J, Liu A (2016). The incidence of post-traumatic stress disorder among survivors after earthquakes: a systematic review and meta-analysis. BMC Psychiatry.

[6] American Psychiatric Association. *Diagnostic and Statistical Manual of Mental Disorders, First Edition (DSM-5)*. Washington, D.C.: APA, 2013. Trad. it.: *DSM-5. Manuale diagnostico e statistico dei disturbi mentali. Quinta edizione.* Milano: Raffaello Cortina, 2014

[7] Nussbaum AM (2014). L’esame diagnostico con il DSM-5.

[8] Li X, Aida J, Hikichi H, Kondo K, Kawachi I (2019). Association of post disaster depression and post traumatic stress disorder with mortality among older disaster survivors of the 2011 great East Japan earthquake and tsunami. JAMA Netw Open.

[9] Pistoia F, Conson M, Carolei A, Dema MG, Splendiani A, Curcio G, et al. Post-earthquake distress and development of emotional expertise in young adults. Front Behav Neurosci. 2018;12:91. 10.3389/fnbeh.2018.00091. 10.3389/fnbeh.2018.00091 PMC5951935 29867392

[10] Tang B, Deng Q, Glik D, Dong J, Zhang L. A meta-analysis of risk factors for post-traumatic stress disorder (PTSD) in adults and children after earthquakes. Int J Environ Res Public Health. 2017;14:E1537. 10.3390/ijerph14121537. 10.3390/ijerph14121537 PMC5750955 29292778

[11] Cofini V, Carbonelli A, Cecilia MR, Binkin N, di Orio F (2015). Post traumatic stress disorder and coping in a sample of adult survivors of the Italian earthquake. Psychiatry Res.

[12] D’Argenio P., Carbonelli A., Cofini V., Diodati G., Gigantesco A., Granchelli C., Luzi P., Mancini C., Minardi V., Mirante N., Tarolla E., Trinito M.O., Bella A. & Salmaso S. *Risultati dello studio CoMeTeS (Conseguenze a Medio Termine del Sisma): stato di salute della popolazione dopo il terremoto del 2009 in Abruzzo.* Roma: Istituto Superiore di Sanità. (Rapporti ISTISAN 13/2), 2013.

[13] Dell’Osso L, Carmassi C, Massimetti G, Conversano C, Daneluzzo E, Riccardi I, et al. Impact of traumatic loss on post-traumatic spectrum symptoms in high school students after the L’Aquila 2009 earthquake in Italy. J Affect Disord. 2011a;134(1–3):59–64. 10.1016/j.jad.2011.06.025. 10.1016/j.jad.2011.06.025 21803426

[14] Dell’Osso L, Carmassi C, Massimetti G, Stratta P, Riccardi I, Capanna C, et al. Age, gender, and epicenter proximity effects on post-traumatic stress symptoms in L’Aquila 2009 earthquake survivors. J Affect Disord. 5. 2013;146(2):174–80. 10.1016/j.jad.2012.08.048. 10.1016/j.jad.2012.08.048 23098626

[15] Carmassi C, Stratta P, Massimetti G, Bertelloni CA, Conversano C, Cremone IM, et al. New DSM-5 maladaptive symptoms in PTSD: gender differences and correlations with mood spectrum symptoms in a sample of high school students following survival of an earthquake. Ann Gen Psychiatry. 2014;18:13–28. 10.1186/s12991-014-0028-9. 10.1186/s12991-014-0028-9 PMC4322820 25670961

[16] Dell’Osso L, Carmassi C, Massimetti G, Daneluzzo E, Di Tommaso S, Rossi A (2011). Full and partial PTSD among young adult survivors 10 months after the L’Aquila 2009 earthquake: gender differences. J Affect Disord.

[17] Lecic-Tosevski D, Pejuskovic B, Miladinovic T, Toskovic O, Priebe S (2013). Posttraumatic stress disorder in a Serbian community seven years after trauma exposure. J Nerv Ment Dis.

[18] Priebe S, Grappasonni I, Mari M, Dewey M, Petrelli F, Costa A (2009). Posttraumatic stress disorder six months after an earthquake: findings from a community sample in a rural region in Italy. Soc Psychiatry Psychiatr Epidemiol.

[19] Bianchini V, Roncone R, Giusti L, Casacchia M, Cifone MG, Pollice R (2015). PTSD growth and substance abuse among a college student community: coping strategies after 2009 l’Aquila earthquake. Clin Pract Epidemiol Ment Health.

[20] Valenti M, Masedu F, Mazza M, Tiberti S, Di Giovanni C, Calvarese A (2013). A longitudinal study of quality of life of earthquake survivors in L’Aquila, Italy. BMC Public Health.

[21] Mollica FR, Caspi-Yavin Y, Lavelle J, Tor S, Chan TYS, Pham T, Ryan A, de Marneffe D (1996). Harvard trauma questionnaire (manual). TORTURE Supplementum.

[22] Barrios Suarez E (2013). Two decades later: the resilience and post-traumatic responses of indigenous Quechua girls and adolescents in the aftermath of the Peruvian armed conflict. Child Abuse & Neglect, 37.

[23] De Fouchier C, Blanchet A, Hopkins W, Bui E, Ait-Aoudia M, Jehel L (2012). Validation of a French adaptation of the Harvard trauma questionnaire among torture survivors from sub-Saharan African countries. Eur J Psychotraumatol.

[24] Housen T, Lenglet A, Ariti C, Ara S, Shah S, Dar M, Hussain A, Paul A, Wagay Z, Viney K, Janes S, Pintaldi G (2018). Validation of mental health screening instruments in the Kashmir Valley, India. Transcult Psychiatry.

[25] Lhewa D, Banu S, Rosenfeld B, Keller A (2007). Validation of a Tibetan translation of the Hopkins symptom Checklist-25 and the Harvard trauma questionnaire. Assessment.

[26] Vukčević M, Momirović J, Purić D (2016). Adaptation of Harvard trauma questionnaire for working with refugees and asylum seekers in Serbia. Psihologija.

[27] Vindbjerg E, Carlsson J, Mortesense EL, Elklit A, Makransky G (2016). The latent structure of posttraumatic stress disorder among Arabic-speaking refugees receiving psychiatric treatment in Denmark. BMC Psychiatry.

[28] Shevlin M, Elklit A (2012). The latent structure of posttraumatic stress disorder: different models or different populations?. J Abnorm Psychol.

[29] Sousa V, Rojjanasrirat W (2010). Translation, adaptation and validation of instruments or scales for use in cross-cultural health care research: a clear and user friendly guideline. J Eval Clin Pract.

[30] Mollica RF, McDonald LS, Massagli MP, Silove D (2004). Measuring trauma, measuring torture: instructions and guidance on the utilization of the Harvard program in refugee Trauma’s versions of the Hopkins symptom Checklist-25 (HSCL-25), the Harvard trauma questionnaire (HTQ) Harvard program in refugee trauma.

[31] Bradley, A.P., & Longstaff I.D. Sample size estimation using the receiver operating characteristic curve. In proceedings of the 17th International Conference on Pattern recognition, 2004, 428–431.

[32] Metz CE (1978). Basic principles of ROC analysis. Semin Nucl Med.

[33] Shoeb M, Weinstein H, Mollica R (2007). The Harvard trauma questionnaire: adapting a cross-cultural instrument for measuring torture, trauma, and posttraumatic stress disorder in Iraqi refugees. Int J Soc Psychiatry.

[34] Valenti M, Fujii S, Kato H, Masedu F, Tiberti S, Sconci V (2013). Validation of the Italian version of the screening questionnaire for disaster mental health (SQD) in a post-earthquake urban environment. Ann Ist Super Sanita.

[35] Blake DD, Weathers FW, Nagy LM, Kaloupek DG, Klauminzer G, Charney DS, et al. A clinician rating scale for assessing current and lifetime PTSD: the CAPS-1. Behav Ther. 1990;13:187–8.

[36] Grupski AE, Hood MM, Hall BJ, Azarbad L, Corsica J (2013). Utility of the binge eating scale in screening for binge eating disorder with bariatric surgery candidates. Obes Surg.

[37] Marcus MD, Wing RR, Hopkins J (1988). Obese binge eaters: affect, cognitions, and response to behavioral weight control. J Consult Clin Psychol.

[38] Celio AA, Wilfley DE, Crow SJ, Mitchell J, Walsh BT (2004). A comparison of the binge eating scale, questionnaire for eating and weight patterns-revised, and eating disorder examination questionnaire with instructions with the eating disorder examination in the assessment of binge eating disorder and its symptoms. Int J Eat Disord.

[39] First MB (2002). Structured clinical interview for DSM-IV-TR axis I disorders - patient edition (SCIDI/ P).

[40] Rosseel, Y. Lavaan: an R package for structural equation modeling. J Stat Softw*,* 2012, 48(2), 1–36. http://www.jstatsoft.org/v48/i02/.

[41] Fox, J., Nie, Z., & Byrnes, J. Sem: structural equation models. R package version 3, 2020, 1-11*.* https://CRAN.R-project.org/package=sem

[42] Haynes S., Smith, G. & Hunsley, J. Scientific foundations of clinical assessment. Scientific Foundations of Clinical Assessment. 2011. 10.4324/9780203829172.

[43] Salkind, N.J. Tests and measurement for people who (think they) hate tests and measurement *(2nd Ed)*. Los Angeles, CA: Sage Publications, 2012.

[44] Cortina JM (1993). What is coefficient alpha? An examination of theory and applications. J Appl Psychol.

[45] Gardner PL (1995). Measuring attitudes to science: Unidimensionality and internal consistency revisited. Res Sci Educ.

[46] Schmitt N (1996). Uses and abuses of coefficient alpha. Psychol Assess.

[47] Taber KS (2018). The use of Cronbach’s alpha when developing and reporting research instrument in science education. Res Sci Educ.

[48] Shrout PE, Fleiss JL (1979). Intraclass correlations: uses in assessing rater reliability. Psychol Bull.

[49] Munaro BH, William F (1997). Statistical methods for health care research.

[50] Khan, R.A., & Brandenburger, T. ROCit: performance assessment of binary classifier with visualization*.* R package version 2*.*1*.*1, 2020. https://CRAN.R-project.org/package=ROCit

[51] Barton, K. MuMIn: Multi-Model Inference*.* R package version 1*.*43*.*17, 2020*.* https://CRAN.R-project.org/package=MuMIn

[52] Zweig MH, Campbell G (1993). Receiver-operating characteristic (ROC) plots: a fundamental evaluation tool in clinical medicine. Clin Chem.

[53] Tremblay J, Pedersen D, Errazuris C (2009). Assessing mental health outcomes of political violence and civil unrest in Peru. Int J Psychiatry.

[54] O’Connell E, Abbott RP, White RS (2017). Emotions and beliefs after a disaster: a comparative analysis of Haiti and Indonesia. Disasters..

[55] Dell’Osso L, Carmassi C, Rucci P, Conversano C, Shear MK, Calugi S (2009). A multidimensional spectrum approach to post-traumatic stress disorder: comparison between the structured clinical interview for trauma and loss Spectrum (SCI-TALS) and the self-report instrument (TALS-SR). Compr Psychiatry.

[56] Pollice, R., Bianchini, V., Roncone, R. & Casacchia, M. Psychological distress and post-traumatic stress disorder (PTSD) in young survivors of L’Aquila earthquake. Rivista di Psichiatria. 2012:47(1):59-64. 10.3411/292. 10.1708/1034.11292 22358218

[57] Pino O (2017). Ricucire i ricordi. La memoria, i suoi disturbi, evidenze di efficacia dei trattamenti riabilitativi.

[58] Dell’osso L, Carmassi C, Stratta P, Massimetti G, Akiskal KK, Akiskal HS, et al. Gender differences in the relationship between maladaptive behaviors and post-traumatic stress disorder. A study on 900 l’ Aquila 2009 earthquake survivors. Front Psychiatry. 2012;3:111. 10.3389/fpsyg.2012.00111. 10.3389/fpsyt.2012.00111 PMC3537190 23293608

[59] Eytan A, Gex-Fabry M, Toscani L, Bouvier PA (2004). Determinants of post conflict symptoms in Albanian Kosovars. J Nerv Ment Dis.

[60] Prati G, Pietrantoni L. Crescita post-traumatica: Un’opportunità dopo il trauma? [post-traumatic growth: an opportunity after the trauma?]. Psicoter Cogn e Comportamentale. 2006;12(2):133–44.

[61] Berthold SM, Mollica RF, Silove D, Tay K, Lavelle J, Lindert J (2018). The HTQ-5: revision of the Harvard trauma questionnaire for measuring torture, trauma and DSM-5 PTSD symptoms in refugee populations. Eur J Pub Health.

